# Quantifying Huntingtin Protein in Human Cerebrospinal Fluid Using a Novel Polyglutamine Length-Independent Assay

**DOI:** 10.3233/JHD-220527

**Published:** 2022-08-30

**Authors:** Valentina Fodale, Roberta Pintauro, Manuel Daldin, Maria Carolina Spiezia, Douglas Macdonald, Alberto Bresciani

**Affiliations:** a Department of Translational and Discovery Research, IRBM S.p.A., Pomezia, Roma, Italy; b CHDI Management/CHDI Foundation, Los Angeles, CA, USA

**Keywords:** Biomarker, huntingtin, Huntington’s disease, immunoassay, pharmacodynamic

## Abstract

**Background:**

The use of biomarkers has become a major component of clinical trial design. In Huntington’s disease (HD), quantifying the amount of huntingtin protein (HTT) in patient cerebrospinal fluid (CSF) has served as a pharmacodynamic readout for HTT-lowering therapeutic approaches and is a potential disease progression biomarker. To date, an ultrasensitive immunoassay to quantify mutant HTT protein (mHTT) has been used, but additional assays are needed to measure other forms of HTT protein.

**Objective:**

We aimed to develop an ultrasensitive immunoassay to quantify HTT protein in a polyglutamine length-independent manner (mHTT and non-expanded wild type HTT combined) in control and HD participant CSF samples.

**Methods:**

An ultrasensitive, bead-based, single molecule counting (SMC) immunoassay platform was used for the detection of HTT protein in human CSF samples.

**Results:**

A novel ultrasensitive SMC immunoassay was developed to quantify HTT protein in a polyglutamine length-independent manner and shown to measure HTT in both control and HD participant CSF samples. We validate the selectivity and specificity of the readout using biochemical and molecular biology tools, and we undertook a preliminary analytical qualification of this assay to enable its clinical use. We also used this novel assay, along with the previously described mHTT assay, to analyze CSF from control and HD participants. The results of this preliminary set suggests that correlation is present between mHTT and the polyglutamine length-independent HTT levels in human CSF.

**Conclusion:**

We have developed a novel ultrasensitive immunoassay that is able to quantify HTT protein in a polyglutamine length-independent manner in control and HD participant CSF.

## INTRODUCTION

Huntington’s disease (HD) is a neurodegenerative condition caused by the expansion of a CAG-repeat domain in exon 1 of the *HTT* gene [1] that leads to the expression of mutant huntingtin (mHTT) protein with an expanded polyglutamine sequence, which ultimately causes neuronal death [2]. The monogenic nature of HD has led to the concept of decreasing mHTT levels as the most proximal therapeutic strategy for disease modification [3]. Quantifying mHTT in cerebrospinal fluid (CSF), an accessible biofluid of the central nervous system (CNS), may be informative as both a biomarker for patient stratification and as a pharmacodynamic measure for mHTT-lowering therapeutics [4, 5]. It has been previously reported that mHTT can be detected at femtomolar levels in HD individuals using the single-molecule counting (SMC) Erenna assay employing the antibody pair 2B7 and MW1 (termed the CHDI_HTT_040 assay) [4–6]. There also seems to be a correlation between the amount of mHTT protein in the CSF and the disease stage [4, 5, 7]. The quantification of HTT protein in a polyglutamine length-independent manner (mHTT and non-expanded wildtype HTT together) is expected to overcome some limitations of mHTT detection, namely its sensitivity to protein length, polyglutamine length, and conformation [4, 6, 8–11]. Such an assay will provide a measure of the effect of HTT-lowering interventions on the overall HTT levels in select biosamples. Additionally, a polyglutamine length-independent HTT assay should provide data about the selectivity of the intervention, for example, for therapeutics aimed at allele-selective lowering, as well as possible safety concerns related to the lowering non-expanded HTT (the latter being a topic beyond the scope of the present work). Furthermore, we propose that polyglutamine length-independent HTT quantification could provide a pivotal pharmacodynamic readout for the effect of a non-allele selective intervention in a Phase 1 trial of healthy volunteers since HTT can now for the first time be measured in the CSF of that population. Lastly, it may enable both the measurement of the pharmacodynamic effect and an assessment of the extent of lowering in the periphery to achieve the desired degree of brain penetration of an orally delivered candidate therapeutic.

The SMC assay platform is an ultrasensitive bead-based immunoassay where, upon specific recognition, dye-labeled antibodies are excited by a confocal laser and emit fluorescent light as a readout. We have previously shown that this approach can relatively quantify mHTT (selective detection) and polyglutamine length-independent HTT (mHTT and non-expanded wild type HTT combined detection) in pre-clinical samples [9]. We have also successfully used this technique to relatively quantify mHTT in HD human CSF using the CHDI_HTT_040 assay (2B7-MW1). However, to our knowledge, no bona fide ultrasensitive polyglutamine length-independent HTT quantification assay has been developed for human CSF. In the present work, we set out to develop and qualify a SMC immunoassay that is selective and specific for the combined measurement of mHTT and non-expanded wild type HTT in human CSF. We assessed a set of antibody pairs and qualified the assay for its selectivity for the analyte, inter-and intra-run precision of the measurement and accuracy for the analyte concentration, as well parallelism, dilution linearity, spike recovery, and ultimately demonstrate its ability to measure HTT in a polyglutamine length-independent manner in human CSF samples. We then provide an initial insight on the significance of these measured levels and the correlation with mHTT levels in a small cohort of control and HD research participants at different disease stages.

## MATERIAL AND METHODS

### Human CSF and blood samples

Human CSF and blood samples used for the assay development part were collected from healthy and HD patients, at the University College London (UCL) by Dr E. Wild. Additional CSF samples came from the HDClarity multi-site CSF collection initiative (https://hdclarity.net/). All samples were collected as previously described [2]. All work involving human volunteers was performed in accordance with the Declaration of Helsinki of 1975 and approved by the Central London Research Ethics Committee and all HDClarity sites obtained and maintained local ethical approvals.

In order to perform the analysis, the samples and reference proteins were diluted in artificial CSF (aCSF): Na2HPO4^*^7H2O 1.3 mM, NaH2PO4^*^7H2O 0.2 mM, 150 mM NaCl, 3 mM KCl, 1.4 mM CaCl2, 0.8 mM MgCl2.

### Antibodies and recombinant proteins (calibrators)

The MW1 monoclonal antibody recognizing the expanded polyglutamine (polyQ) HTT sequence was developed by Dr. Paul Patterson [12]. The 2B7 monoclonal antibody to the HTT amino terminus and 4C9 monoclonal antibody to the human HTT proline rich domain were generated and characterized as previously described [13]. Additionally, the D7F7 monoclonal antibody recognizing HTT near residue Proline 1220 was obtained from Cell Signaling Technology and the MAB2166 antibody recognizing the HTT domain of 445–459 amino acids was obtained from Millipore (cat# MAB2166).

The 2B7 antibody was conjugated to magnetic particles (MPs) using the Merck SMC Capture Reagents Labeling kit (cat# 03-0077-02) to a final concentration of 25μg/mg of MPs. The MW1, 4C9, D7F7, and MAB2166 antibodies were conjugated with the SMC Detection Reagents Labeling kit (Merck, cat# 03-0076-02) to a final concentration of 1μg/μl. Conjugated/labeled antibodies were diluted in Sample buffer (Candor, Cat# 105 500), prior to performing the assay.

For these studies recombinant full length human HTT proteins (HTT (1-3144) Q17 and HTT (1-3144) Q46) and a large fragment amino terminal human HTT (1-573) Q45 protein were used [14, 15]. Recombinant reference HTT proteins are available from the Coriell Institute (Camden, USA).

### Cell lines

Primary fibroblast cells collected from a compound heterozygous HD patient (GM01085, CAG expansion: 45/23) and control (GM07532, CAG expansion: 23/19) were obtained from Coriell Institute for Medical Research, Camden, NJ, USA. HEK293T cells were obtained from ATCC (Cat# CRL-11268).

### Quantitative PCR (qPCR)

HTT lowering was carried out with an HTT-specific siRNA from SIGMA (cat# SASI_HS01_00241076) using Lipofectamine 2000 (Invitrogen, cat# 11668019) as the transfection reagent according to the manufacturer’s instruction. HEK293T cells were collected 48 hours after transfection and total RNA was isolated using RNeasy Mini Kit (Qiagen, Cat# 74104) according to the manufacturer’s instructions. To quantify HTT expression levels, one-step qRT-PCR was carried out on 100 ng of RNA using the QuantStudio 12K Flex system (Applied Biosystems) QuantiTect Probe RT-PCR Kit (Qiagen, Cat# 204443) and primers for HTT (IDT, Cat# Hs.PT.58.1696972) and GAPDH (Thermo Fisher, Cat# Hs02758991_g1) according to manufacturer’s instructions. HTT mRNA levels were normalized to the mean of housekeeping gene GAPDH and the fold change was calculated using the 2 -*Δ* *Δ*CT method as described by Livak and Schmittgen [16] using the scrambled treated sample as reference.

### Western blot assay

HEK293T cell cultures were lysed in lysis buffer (TBS, 0.4% Triton X, protease inhibitors cocktail from Roche, Cat# 11836145001), and clarified by centrifugation at 10,000× *g* for 2 min at 4°C. The total protein concentration was determined using the BCA protein assay kit (Thermo Fisher, Cat# 23225) according to manufacturer’s protocol. 30μg of total lysates were denatured at 95 °C in 4x loading buffer (125 mM TrisHCl pH 6.8, 6% SDS, 4 M urea, 4 mM EDTA, 30% glycerol, 4% β-mercaptoethanol, and Bromophenol blue) and loaded on NuPAGE 4–12% Bis-Tris Gel (ThermoFisher). Proteins were transferred to PVDF membranes that were treated for 30 min with TBS, 0.1% Tween, and 0.4% PFA, followed by 1 h blocking in TBS, 0.1% Tween, and 5% not fat milk. The staining with the primary antibodies, MAB2166 (Millipore) and anti-GAPDH (Sigma, cat # G9545), was performed overnight at 4°C in TBS, 0.1% Tween, 5% not fat milk. After washing in in TBS, 0.1% Tween, and 5% non-fat milk, the secondary anti-mouse or anti-rabbit horseradish peroxidase conjugated antibodies, diluted in TBS, 0.1% Tween, and 5% not fat milk, were incubated with the membrane for 1 h at room temperature. Protein bands were revealed using chemiluminescence (ECL from Life Technologies). Images were acquired using a Chemidoc imager (Biorad).

### Single molecule counting immunoassay

The SMC immunoassay was performed as previously described [2] which followed the manufacturer’s instructions utilizing the provided buffers which were not modified. Furthermore, the dilution factor for the capture and detection antibodies were adjusted if necessary, using a bridging experiment when a new antibody conjugation lot was produced. Briefly, the assay starts with a capture step, where the 2B7 conjugated MPs are incubated with samples for 1 h in an Axygen polypropylene V-bottom 96 well plate, previously coated with a solution of 1X SMCTM System Buffer (Merck, Cat# 02-0111-00) 750 mM NaCl, 6% BSA, 0.8% Triton-X, and protease inhibitor cocktail (complete tablets from Roche, Cat# 11836145001). Washes were performed on a magnetic rack, using an Elx405 washer (Biotek), in 1X SMCTM System Buffer. Afterwards MPs were incubated with the dye-conjugated detection antibody (MW1, D7F7, 4C9, or MAB2166) for 1 h. Washes were performed again on a magnetic rack, in an Elx405 washer, in 1X SMCTM System Buffer. The MPs were then transferred into a new Axygen 96 well plate to eliminate nonspecific binding to the plastic. After buffer aspiration the elution buffer (acidic glycine solution, 0.1 M, pH 2.7) was added to the plate for five minutes of incubation under shaking. The eluted detection antibody was transferred to a Nunc 384-well analysis plate and neutralized with neutralization buffer (Tris, 1 M, pH 9). The plate was spun down, sealed, and subsequently analyzed with the Erenna Immunoassay System (Merck). Unique identification numbers have been given to each of these SMC Erenna assays, namely: CHDI_HTT_040 (2B7-MW1), CHDI_HTT_041 (2B7-4C9), CHDI_HTT_042 (2B7-MAB2166), and CHDI_HTT_143 (2B7-D7F7).

### Other assays

Hemoglobin A (HbA) quantification was performed using a commercial ELISA (Bethyl Laboratories, cat# E88-134) according to the manufacturer’s specification.

### Immunodepletion method

Fibroblast cell cultures were lysed in lysis buffer (TBS, 0.4% Triton X, protease inhibitors cocktail from Roche, Cat# 11836145001), and clarified by centrifugation at 10,000× *g* for 2 min at 4°C. The total protein concentration was determined using the BCA protein assay kit (Thermo Fisher, Cat# 23225) according to manufacturer’s protocol. 200μg of fibroblasts total lysates or 800μl of human CSF were pre-cleared on naked protein G Dynabeads (Invitrogen, cat# 10003D) for 1 h on a rotating wheel at room temperature. HTT was immunoprecipitated for pre-cleared samples using the MW1 or the MAB2166 antibodies captured on protein G Dynabeads (2μg of antibody for 20μl of beads solution) following manufacturer’s instructions. The pre-cleared and immuno-depleted samples were tested for polyglutamine length-independent (mHTT and non-expanded wild type HTT combined) HTT quantitation by SMC assay according to the above protocol.

### Data analysis

Data analysis was performed using GraphPad Prism software in order to obtain standard curve fitting and back-calculations on fitting models. The standard curve was obtained without associating any weight to each standard concentration.

## RESULTS

### Testing of HTT polyglutamine length-independent antibodies

**Fig. 1:**
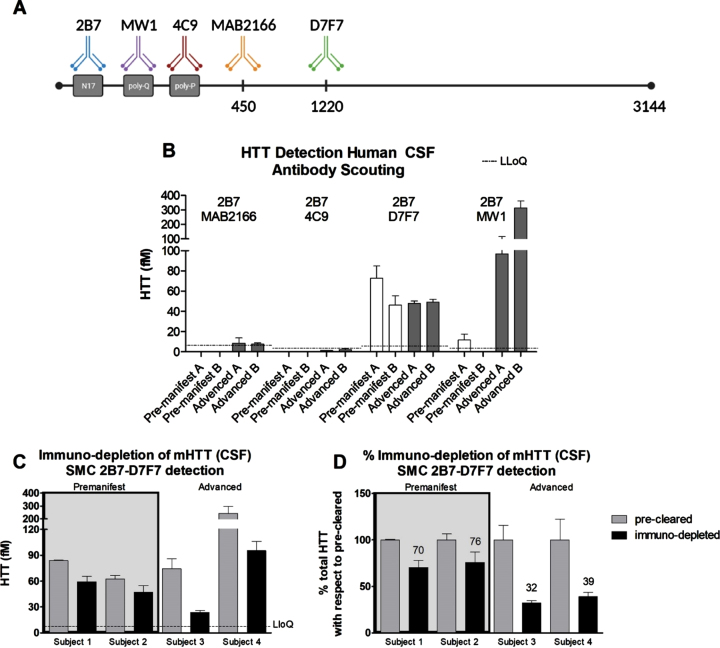
A) Schematic of the anti-HTT antibodies used in the present work. The amino acid position of each antigenic region is not properly scaled to allow for a better depiction of the scheme. B) Polyglutamine length-independent (mHTT and non-expanded wild type HTT combined) HTT quantification using the SMC technology and the antibody pairs indicated in each panel (top, capture; bottom, detection) with white bars representing pre-manifest HD CSF samples and black bars representing advanced HD CSF samples. Assay identification numbers: CHDI_HTT_040 (2B7-MW1), CHDI_HTT_041 (2B7-4C9), CHDI_HTT_042 (2B7-MAB2166), and CHDI_HTT_143 (2B7-D7F7). The HTT (1-573) Q45 calibrator was used for the CHDI_HTT_040 (2B7-MW1) SMC assay while the FL HTT (1-3144) Q46 was used as calibrator for all other assays. Data are reported as the average and standard deviation of three technical replicates. C) Immuno-depletion of mHTT by MW1 in premanifest and advanced HD participant CSF samples (FL Q46 HTT was used as calibrator). Grey bars represent the protein G pre-cleared samples and black bars represent the mHTT immuno-depleted samples. All samples are reported as the average and standard deviation of three technical replicates. D) The results reported in panel B are presented as percentage with respect to the pre-cleared samples. Concentrations reported in panel C and D are relative quantitative values.

In order to develop a SMC assay able to quantify HTT in human CSF in a polyglutamine-length independent manner, we set out to identify antibodies suited to this aim. Due to limitations in the availability of human HD CSF at the time this work was carried out, we had to make some initial assumptions. First, we fixed the 2B7 antibody, directed against the N17 portion of HTT, as the capture antibody. This strategy was previously published by us and others to be successful [4, 6, 9, 17–19]. Furthermore, the 2B7 antibody [13] has been used as the SMC capture antibody in combination with MW1 in the CHDI_HTT_040 assay for mHTT quantification in clinical trials [20]. For the detection antibodies, we chose the more widely used or previously characterized from our work [9], among these are: 4C9, MAB2166, and D7F7 (Fig. 1A). To identify the optimal antibody combination pair for assay development, we assessed them for the detection of HTT in two pre-manifest and two advance HD CSF samples. As positive control, we tested these HD CSF samples in the standard mHTT quantification CHDI_HTT_040 assay (2B7-MW1) [6] using HTT (1-573) Q45 protein as the reference standard calibrator. As expected, we were able to detect a significant signal in the advanced HD CSF samples, but for the pre-manifest HD samples the signal was close or below lower limit of quantification (LLoQ) (Fig. 1B). For the novel polyglutamine length-independent HTT assay antibody pairs, we used the full length (FL) HTT (1-3144) Q46 as reference standard calibrator. Among the tested pairs, only the CHDI_HTT_143 (2B7-D7F7) assay was able to produce signals well above the assay LLoQ (Fig. 1B), thus this antibody combination was selected for further validation and qualification.

### Selectivity validation via mutant HTT immuno-depletion in HD CSF

In order to ensure that the signal we detect in human CSF using the CHDI_HTT_143 SMC 2B7-D7F7 assay is indeed attributable to HTT, we set out to validate the assay by immuno-depletion of HTT in human CSF. To this aim, we excluded the use of the antibodies also present in the quantification assay (2B7 and D7F7) to avoid confounds. Among the remaining antibodies considered, the 4C9 and the MAB2166 antibodies were also excluded given that they were not found effective when used for detection. As a consequence, we used MW1 for the preferential binding to mHTT with respect to wild type HTT and/or other polyglutamine containing proteins. This point is debated as the MW1 selectivity for mHTT can be influenced by a number of factor such as poly-glutamine repeat length and HTT proteoforms, but here we used MW1 for the immuno-depletion of mHTT in human HD CSF as a means for the preliminary validation of the specificity of the CHDI_HTT_143 (2B7-D7F7) polyglutamine length-independent assay, whereas studies for the assessment of the MW1 selectivity are outside the scope of the present work. Four HD CSF samples were selected among those with moderate to high CHDI_HTT_143 (2B7-D7F7) assay measured HTT concentrations (∼50–100 fM) to ensure the detection of the anticipated HTT signal decrease. Specifically, two pre-manifest and two advanced HD participant CSF samples were used. Based on the previously reported findings that mHTT levels are higher in later stage HD individuals [4, 5], we deliberately selected these samples expecting a higher degree of immuno-depletion in advanced subjects than in pre-manifest. It should be noted that the higher levels of mHTT as measured by the CHDI_HTT_040 (2B7-MW1) SMC assay in the CSF of advanced HD research participants does not necessarily mean a higher full-length mHTT concentration in CSF but may or may not be related to changes of mHTT proteoforms with improved binding by the MW1 antibody in that particular assay. In this experiment, the MW1 antibody was immobilized to protein G beads, while naked protein G beads were used to pre-clear the samples. The precleared and the immuno-depleted samples were then assayed via the CHDI_HTT_143 (2B7-D7F7) SMC assay. Figure 1C shows that the immuno-depletion was able to decrease polyglutamine length-independent HTT suggesting that this assay is a *bona fide* HTT measure in human CSF. And in line with expectations, the relative decrease of the CHDI_HTT_143 (2B7-D7F7) assay measured HTT (mHTT and non-expanded wild type HTT combined) was consistent within pre-manifest and advanced HD participant CSF samples, but different in magnitude with the former decrease being 20–30% while the latter was 70–80% (Fig. 1D) being due to the greater amount of mHTT in advanced stage HD participant CSF and the mHTT selectivity of the MW1 antibody used for immuno-depletion.

### Selectivity validation in vitro

**Fig. 2:**
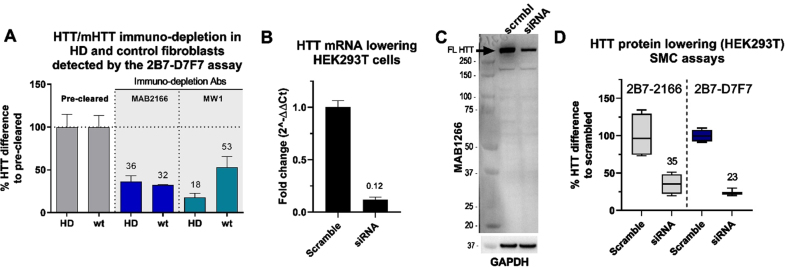
A) CHDI_HTT_143 (2B7-D7F7) SMC assay HTT quantification in mHTT or HTT depleted samples. The quantification was carried out by the CHDI_HTT_143 (2B7-D7F7) SMC assay against the FL Q46 HTT as calibrator. The wild type (wt) fibroblasts were the GM07532 (CAG expansion: 23/19), while the HD fibroblasts (HD) were the GM01085 (CAG expansion: 45/23). All samples are reported as the average and standard deviation of three technical replicates. B) HTT mRNA lowering in HEK293T cells. The fold change of the HTT mRNA levels are reported using the scrambled sample as reference. C) Representative Western blot with full length HTT (arrow) recognized by the MAB2166 antibody, after scrambled siRNA or HTT siRNA treated HEK293T cells. D) HTT levels detected by the CHDI_HTT_042 (2B7-MAB2166) SMC assay or the CHDI_HTT_143 (2B7-D7F7) SMC assay on scrambled or HTT siRNA treated HEK293T cells. All samples are reported as the average and standard deviation of three technical replicates.

To further validate the selectivity of the CHDI_HTT_143 (2B7-D7F7) SMC assay, we assessed it using HTT-depleted human fibroblast cell lysates. To this aim, we used one HD fibroblast line (GM01085; CAG 45/23) and a non-HD control fibroblast line (GM07532; CAG 23/19). Again, we used the MW1 to pull-down mHTT from the HD fibroblast lysates and in non-HD fibroblast as a control. In addition, we used the MAB2166 to immuno-deplete both mHTT and non-expanded wildtype HTT in both lines. The MAB2166 was previously reported to be a polyglutamine length-independent specific anti-HTT antibody [9, 14]. It has to be noted that the immuno-depletion procedure with both MW1 and MAB2166 was intended to assess the selectivity of the CHDI_HTT_143 (2B7-D7F7) SMC assay and is not sufficiently optimized to draw conclusions on the MW1 or MAB2166 specificity. Nevertheless, as expected, the CHDI_HTT_143 (2B7-D7F7) SMC assay showed a consistent 64–68% reduction in HTT signal in both HD and non-HD fibroblasts after MAB2166 immuno-depletion (Fig. 2A). Alternatively, the MW1 immuno-depletion resulted in a preferential reduction of the signal in the HD fibroblasts (82%) when comparted to the non-HD fibroblasts (47%) as calculated by the percent HTT difference to pre-cleared (Fig. 2A). To further demonstrate the CHDI_HTT_143 (2B7-D7F7) SMC assay selectivity, we lowered HTT in wildtype HEK293T cells via siRNA and used scrambled siRNA as a control (Fig. 2B). This HTT lowering effect was detected by the CHDI_HTT_143 (2B7-D7F7) SMC assay (Fig. 2D) and was comparable to the reduction detected via western blot (Fig. 2C), approximately 70%. The CHDI_HTT_143 (2B7-D7F7) assay detection was found to be less variable than the CHDI_HTT_042 (2B7-MAB2166) assay (Fig. 2D). The present results taken all together qualify the CHDI_HTT_143 (2B7-D7F7) SMC assay as a selective measure of soluble HTT in a polyglutamine-length independent manner (mHTT and non-expanded wild type HTT combined detection).

### Sensitivity and HTT form selectivity

**Fig. 3:**
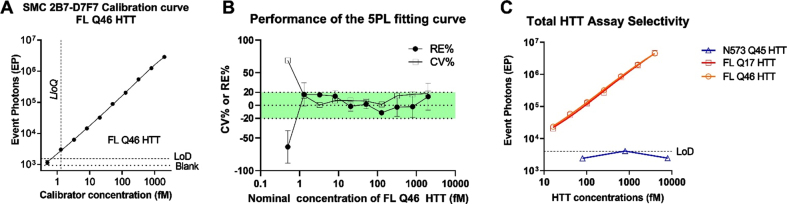
A) Calibration curve of the CHDI_HTT_143 (2B7-D7F7) SMC assay. The FL Q46 HTT was used as calibrator for the CHDI_HTT_143 (2B7-D7F7) SMC assay, and the points were fitted with a 5LP logistic regression. The limit of detection (LoD) was calculated as the average of blanks plus three standard deviations. All values are reported as the average and standard deviation of three technical replicates. B) CV% and RE% of the calculated concentrations with respect to the nominal ones for all calibrator concentrations. The green shaded area represents the acceptance criteria zone (±20% RE% and CV%). All values are reported as the average and standard deviation of three technical replicates. C) Polyglutamine length-independence and FL HTT selectivity of the CHDI_HTT_143 (2B7-D7F7) SMC assay; the N573 Q45 HTT calibrator was used at three representative concentrations spanning the dynamic range. All samples are reported as the average and standard deviation of three technical replicates.

To determine the sensitivity of the CHDI_HTT_143 (2B7-D7F7) SMC assay, a standard curve was generated using full length HTT (1-3144) Q46 (FL Q46). A serial dilution was generated by spiking 2000 fM (703.7 pg/ml) of recombinant protein into aCSF used as surrogate matrix due to our previous work demonstrating that aCSF is a viable matrix surrogate for the standard curve [6]. The standard curve was freshly prepared from single-use aliquots of FL Q46. Each concentration was run in triplicate and a zero-reference standard concentration was prepared in aCSF and used as blank. A concentration-response relationship was generated and fitted with a five-parameter logistic (5PL) model (Fig. 3A) [21], and the LLoQ was determined to be 1.3 fM. The analyte concentrations, back-calculated by fitting model, were compared to the nominal ones to determine the relative error percentage (% RE) and the coefficient of variation percentage (% CV). Both % RE and the % CV should not exceed 20% (25% at LLoQ) as indicated by the FDA guidance for ligand binding assay (LBA) validation [22]. The majority of the dilution points (nine out of ten points) are compliant with the acceptance criteria (Fig. 3B). We then explored the selectivity of the assay for the full length HTT proteins and its polyglutamine-length independence. To this aim, three variants of human recombinant HTT proteins were tested: HTT (1-573) Q46, FL HTT Q17, and FL HTT Q46. All proteins were diluted from 2 pM to 15 fM for seven non-zero dilutions in aCSF. The results (Fig. 3C) showed that the assay detected FL HTT Q17 and FL mHTT Q46 similarly thereby demonstrating the polyglutamine length-independent nature of the CHDI_HTT_143 (2B7-D7F7) SMC assay. In contrast, the N573 HTT Q45 protein was not detected as expected (all dilutions were below the assay LOD) due to the lack of the D7F7 epitope in this HTT 1-573 fragment (Fig. 3C).

### 3.5 Inter- and intra-run precision and accuracy of the calibration curve

The FDA guidelines request that precision and accuracy be determined using replicate analysis of samples containing endogenous detectable amounts of the analyte (QC samples) [22]. Due to the limited availability of a sufficient amount of human CSF samples, Intra-run precision and accuracy evaluation was carried out by testing six technical replicates of each of the seven FL HTT Q46 standard concentrations for six independent runs. Inter-run evaluation was performed by determining the precision and accuracy among six independent runs. The concentrations of the calibration curve were selected based on the expected range of mutant and non-expanded wild type combined HTT concentration in human CSF (> 10 fM). Precision was calculated as % CV between the six technical replicates for each concentration within each run and between the mean of different runs (< 25% was set as acceptance criterion). Accuracy was calculated as % RE between the concentrations, back-calculated on the standard curve itself for each standard technical point, and the nominal concentration of that point within each run and between the mean concentrations of different runs (< 25% was set as acceptance criterion). Intra-run accuracy and precision were achieved according to the established acceptance criteria for all the points with the exception of the CV% value at the lowest HTT concentration in run #3 (Table 1). The inter-run accuracy and precision were achieved for all standard concentrations tested according to the established acceptance criteria (Table 2).

**Table 1:** Intra-run precision (top) and accuracy (lower) results of the CHDI_HTT_143 (2B7-D7F7) SMC assay

	CV %					
nominal fM	N	Run 1	Run 2	Run 3	Run 4	Run 5	Run 6
4000	6	4.6	10.6	14.4	2.2	1.7	16.8
1600	6	3.7	15.6	19.9	15.8	17.8	17.2
640	6	10.1	13.3	17.2	4.0	7.1	11.3
256	6	12.8	12.1	11.5	8.5	0.1	15.3
102	6	13.0	12.8	3.8	4.2	10.8	13.1
41	6	6.3	15.3	19.2	8.4	12.4	11.2
16	6	13.0	13.3	26.3	8.4	15.0	24.9
	RE %					
nominal fM	N	Run 1	Run 2	Run 3	Run 4	Run 5	Run 6
4000	6	–0.2	5.5	–0.2	0.8	4.0	3.0
1600	6	3.7	–7.4	3.5	0.0	–3.5	–2.3
640	6	–3.2	4.0	–1.3	4.5	2.3	4.1
256	6	2.3	–4.2	0.8	–9.4	2.9	–1.2
102	6	0.1	11.6	1.1	3.8	–0.3	3.0
41	6	2.1	–3.7	0.3	10.9	–4.5	–1.1
16	6	–1.0	16.5	1.9	–4.8	4.5	–4.7

**Table 2:** Inter-run precision and accuracy results of the CHDI_HTT_143 (2B7-D7F7) SMC assay

	curve point	nominal fM	PLATE 1 average fM	PLATE 2 average fM	PLATE 3 average fM	PLATE 4 average fM	PLATE 5 average fM	PLATE 6 average fM	6 plates average fM	SD	CV(%)	RE(%)	*N*
	**1**	4000	3990.8	4220.4	3993.1	4030.6	4159.3	4123.4	4086.3	95.5	2.3	2.1	6
	**2**	1600	1659.2	1481.2	1656.3	1601.0	1543.4	1562.3	1583.9	69.0	4.3	–1.0	6
Inter-run	**3**	640	619.6	665.4	631.5	669.1	654.9	666.0	651.1	20.7	3.2	1.7	6
Precision and	**4**	256	261.9	245.1	258.1	231.8	263.5	252.8	252.2	12.0	4.8	–1.5	6
Accuracy	**5**	102	102.5	114.3	103.6	106.3	102.0	105.4	105.7	4.5	4.3	3.2	6
	**6**	41	41.8	39.4	41.1	45.4	39.1	40.5	41.2	2.3	5.6	0.7	6
	**7**	16	16.2	19.1	16.7	15.6	17.1	15.6	16.7	1.3	7.8	2.0	6

### Parallelism, dilution linearity, and spike recovery

**Fig. 4:**
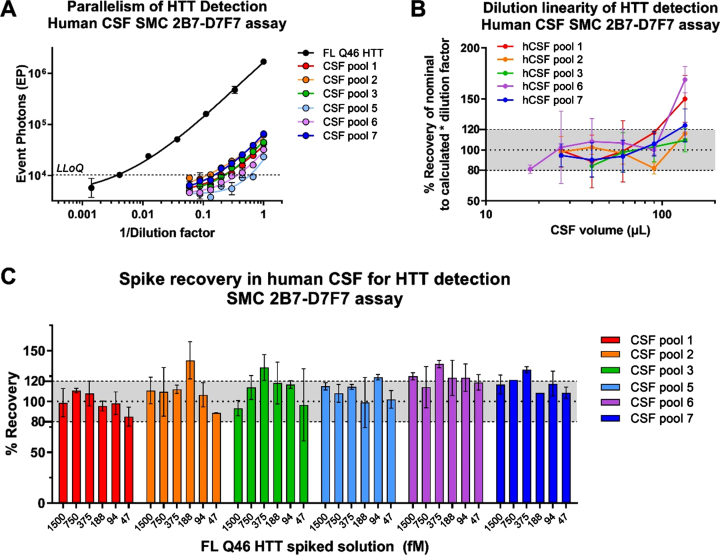
A) Parallelism of the CHDI_HTT_143 (2B7-D7F7) SMC assay in healthy human CSF. On the x-axis the inverse of dilution factor, with respect to the highest volume used, is reported. For the FL Q46 HTT calibrator the inverse of the dilution factor is calculated with respect to the highest concentration tested. All samples are reported as the average and standard deviation of three technical replicates. B) Dilution linearity of CHDI_HTT_143 (2B7-D7F7) SMC assay HTT detection in healthy human CSF. On the y-axis, the back-calculated concentrations of each dilution, multiplied for the dilution factor with respect to undiluted, is used to calculate the RE% with respect to the average concentration of all dilutions within one participant. All samples are reported as the average and standard deviation of three technical replicates. C) FL Q46 HTT spike recovery in healthy human CSF detected via the CHDI_HTT_143 (2B7-D7F7) SMC assay. The recovery percentage is the ratio between the back-calculated concentration minus the nominal concentration and the nominal concentration (x100). All samples are reported as the average and standard deviation of three technical replicates.

The parallelism assessment is used to demonstrate that the endogenous analyte, in the biological matrix of interest, behaves in a similar immunochemical manner to the standard protein in the same or similar matrix [9]. In the present work, parallelism was assessed between the calibration standard curve and six human CSF sample pools collected from healthy participants. CSF samples were serially diluted in aCSF of a factor of 1.5 to obtain eight dilutions. The signals obtained from analysis of all CSF samples were above the LLoQ of the assay for at least three dilutions with the exception of CSF pool 5 (Fig. 4A). Parallelism was evaluated by fitting CSF serial dilution signals with the 5PL model by sharing all parameters among the curves with the exception of EC50 and Slope. R2 values were between 0.87 and 0.99. Slopes were similar among the curves obtained (CV%  < 20%, excluding CSF pool 5) indicating that the sample response curves are parallel to the reference curve and possible matrix effects do not appear to be relevant in analyte detection in human CSF. With regard to dilution linearity, the HTT concentration in fM at each sample dilution point, was back-calculated on the reference standard curve and multiplied by the dilution factor. Dilution linearity was verified by calculating the recovery % between the concentration (fM) obtained from each point, above the LLOQ, and the average concentration fM among all the diluted points within a sample. The recovery percentage among the dilution points was included between 80 and 120% for all samples below 90μL of CSF (Fig. 4B). CHDI_HTT_143 (2B7-D7F7) SMC assay HTT concentrations at the maximum CSF volume (135μl) were outside the dilution linearity acceptance criteria for four of six samples. In conclusion, the parallelism test performed met the acceptance criteria and CHDI_HTT_143 (2B7-D7F7) SMC assay HTT dilution linearity was demonstrated for volumes below 90μl of human CSF.

Due to the relatively low concentration of HTT in human CSF, the optimum volume to obtain a reliable CHDI_HTT_143 (2B7-D7F7) SMC assay HTT quantification was established to be 90μL, the highest possible within the dilution linearity range. The CHDI_HTT_143 (2B7-D7F7) SMC assay HTT spike recovery study was carried out to evaluate the influence of the CSF matrix in protein detection. The FL HTT Q46 reference standard was spiked at six concentrations (1500 –47 fM) in six pooled human CSF samples from healthy donors. The spike recovery percentage was calculated between the CHDI_HTT_143 (2B7-D7F7) SMC assay determined HTT amount and its nominal value. At least four out of six HTT spiked concentrations were recovered within the accepted range of 80–120% of the nominal values for five out of six CSF pools (Fig. 4C), meeting the acceptance criteria of at least 66.7% of the spiked solutions being properly recovered [21, 23].

### Specificity

**Fig. 5:**
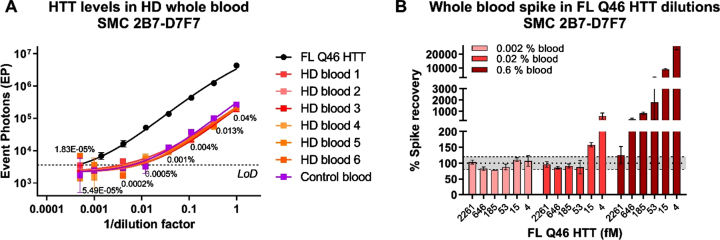
A) CHDI_HTT_143 (2B7-D7F7) SMC assay HTT levels in HD and control participant whole blood. On the x-axis the inverse of dilution factor, with respect to the highest dilution used, is reported. For the FL Q46 HTT calibrator, the inverse of the dilution factor is calculated with respect to the highest concentration tested. The number reported close to each blood dilution is the final percentage of blood diluted in aCSF. All samples are reported as the average and standard deviation of three technical replicates. B) aCSF FL Q46 HTT recovery after spiking three dilutions of human healthy whole blood as detected via the CHDI_HTT_143 (2B7-D7F7) SMC assay. The recovery percentage is the ratio between the back-calculated concentration minus the nominal concentration and the nominal concentration (x100). All samples are reported as the average and standard deviation of three technical replicates.

We have previously shown that mHTT levels in HD human participant CSF are sensitive to blood contamination during sampling, likely due to the higher concentration of mHTT in blood than in CSF [6]. For this reason, we have recommended to check for blood contamination in CSF by using the quantification of hemoglobin (HbA) as a marker and to discard samples with HbA levels greater than 2μg/mL. Here we set out to confirm if that same limit is valid for the CHDI_HTT_143 (2B7-D7F7) SMC assay. We initially determined the levels of HTT in six HD participant whole blood samples and a pool of healthy participants whole blood as control. We found no striking differences between the HD and the healthy pool in terms of CHDI_HTT_143 (2B7-D7F7) SMC assay HTT concentration that averaged 3.5 pM (Fig. 5A) confirming the hypothesis that mHTT/HTT is more concentrated in blood that in CSF by an average factor of 175 (average CHDI_HTT_143 (2B7-D7F7) SMC assay HTT in human CSF is around 20 fM). To investigate the maximal acceptable whole blood contamination limit for no interference in CHDI_HTT_143 (2B7-D7F7) SMC assay HTT detection in human CSF, we generated a serial dilution of FL Q48 HTT protein (from 2261.5 fM to 4.3 fM) in aCSF and then spiked in three dilutions of healthy donor whole blood at 0.6%, 0.02%, and 0.002%, corresponding to simulated contamination of 540, 18, and 1.8μg/mL HbA concentration. Among these whole blood levels, only the 0.002% (1.8μg/mL HbA) allowed the recovery of all the spiked concentrations while 0.02% blood (18μg/mL HbA) allowed the recovery of HTT concentrations higher or equal to 18.6 fM (Fig. 5B). Finally, the spiked HTT concentrations at 0.6% blood (540μg/mL HbA) appeared to be over-recovered (Fig. 5B). These results are consistent with the threshold previously set for the detection of mHTT in human CSF, therefore we recommend setting 2μg/mL HbA as the maximum acceptable blood contamination for the quantitation of HTT via the CHDI_HTT_143 (2B7-D7F7) SMC assay.

### CHDI_HTT_143 (2B7-D7F7) SMC assay HTT and CHDI_HTT_040 (2B7-MW1) SMC assay mHTT levels in human HD participant CSF

We have qualified the CHDI_HTT_143 (2B7-D7F7) SMC assay for detection of polyglutamine length-independent (mHTT and non-expanded wild type HTT combined) HTT in control human CSF. In this next study, we used the CHDI_HTT_143 (2B7-D7F7) and CHDI_HTT_040 (2B7-MW1) SMC assays to quantify polyglutamine length-independent HTT and mHTT, respectively, in a small cohort of human CSF samples from 30 participants including six controls and 24 HD participants spanning a range of disease stages. The aim of this study was to assess and potential variations in CHDI_HTT_143 (2B7-D7F7) SMC assay HTT levels with disease stage and perhaps establish a correlation with mHTT levels in CSF. We first considered the issue related to the reference protein (calibrator) to be used in this study. In fact, for mHTT quantification via the CHDI_HTT_040 (2B7-MW1) SMC assay, it is common practice to use the HTT (1-573) Q45 (N573 Q45; a.k.a. N548 Q45 depending on nomenclature used) as calibrator. As we have shown in the present work, the CHDI_HTT_143 (2B7-D7F7) SMC assay does not detect the short N-terminal protein calibrators as the epitope for the D7F7 antibody is directed to the domain surrounding the proline 1220 residue. As a consequence, in order to make the comparison between CHDI_HTT_143 (2B7-D7F7) SMC assay (mHTT and non-expanded wild type HTT combined) HTT and CHDI_HTT_040 (2B7-MW1) SMC assay mHTT levels as meaningful as possible, we decided to use both the N573 Q45 HTT and the FL Q46 HTT as calibrator proteins for the mHTT assay. It is also important to note that we have previously shown that the full length HTT proteins display decreased sensitivity in the CHDI_HTT_040 (2B7-MW1) SMC mHTT assay compared to the N573 HTT fragment protein [6, 9].

**Fig. 6:**
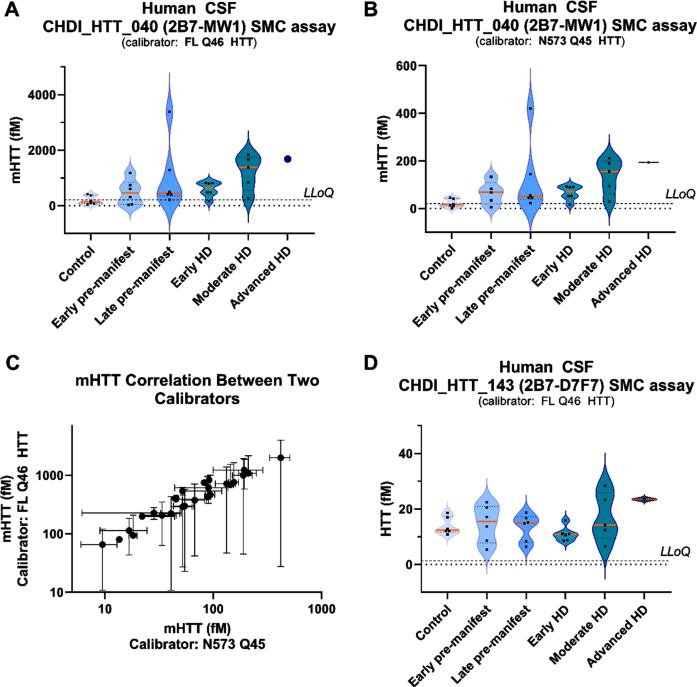
CHDI_HTT_143 (2B7-D7F7) SMC assay HTT and CHDI_HTT_040 (2B7-MW1) SMC assay mHTT levels in control and HD participant CSF samples. For all violin plots, the orange horizontal line represents the median while the dashed horizontal lines represent the upper and the lower quartiles. A) mHTT levels in control and HD participant CSF as measured by the CHDI_HTT_040 (2B7-MW1) SMC assay. Concentrations were calculated against the FL Q46 HTT calibrator and have to be considered as relative quantitative values. All samples are reported as the average and standard deviation of three technical replicates. B) mHTT levels in control and HD participant CSF as measured by the CHDI_HTT_040 (2B7-MW1) SMC assay. Concentrations were calculated against the N573 Q45 HTT calibrator and have to be considered as relative quantitative values. All samples are reported as the average and standard deviation of three technical replicates. C) Correlation between mHTT levels as back-calculated against the N573 Q45 HTT (x-axis) versus the FL Q46 HTT (y-axis). Due to non-Gaussian distribution of both mHTT data sets, as determined by the Shapiro-Wilk test, the correlation test was carried out by a non-parametric Spearman correlation test. *p* = < 0.0001, *r* = 0.95, 95% confidence interval = 0.89 to 0.98. D) HTT levels in control and HD participants as measured by the CHDI_HTT_143 (2B7-D7F7) SMC assay. Concentrations were calculated against the FL Q46 HTT calibrator and have to be considered as relative quantitative values. All samples are reported as the average and standard deviation of three technical replicates. LLoQ are indicated in violin plots: 205 fM for FL Q46 and 20.5 fM for N573 Q45 in CHDI_HTT_040 (2B7-MW1) SMC assay; 1.3 fM for FL Q46 in CHDI_HTT_143 (2B7-D7F7) SMC assay.

Next, we tested the 30 participant CSF samples for HbA and found them all to be below the established threshold of 2μg/mL HbA, thus qualifying them for further testing. The mHTT levels were found to be close or below the LLoQ in all non-HD control CSF samples, while we observed the expected progressive increase in mHTT detection levels with disease stage in the HD participant CSF samples (Fig. 6A) [5]. In addition, according to the above-mentioned decreased performance of the FL Q46 HTT with respect to the HTT N573 Q45 calibrator in the mHTT assay, we did observe a higher back-calculated mHTT concentration in HD participant CSF samples when the FL Q46 HTT was used as a calibrator compared to when the N573 Q45 HTT is used (Fig. 6A, B). It should be noted that as claimed before [6, 9], absolute calculated values of mHTT detected by the 2B7-MW1 SMC assay is a relative measurement and should not be compared across different studies, especially if different calibrators are used. Nonetheless, when we correlated the back-calculated mHTT levels on the FL Q48 HTT and the N573 Q45 mHTT calibrators we observed a strong correlation (*p* = < 0.0001, Fig. 6C). With regard to CHDI_HTT_143 (2B7-D7F7) SMC assay HTT levels in these CSF samples, we found them to be in the range of 5–35 fM (Fig. 6D). CHDI_HTT_143 (2B7-D7F7) SMC assay HTT was detected in control participant CSF samples with no trend related to the HD disease stage except possibly for the advanced stage CSF samples available to us (albeit only one), thus requiring further investigation when samples become available.

**Fig. 7:**
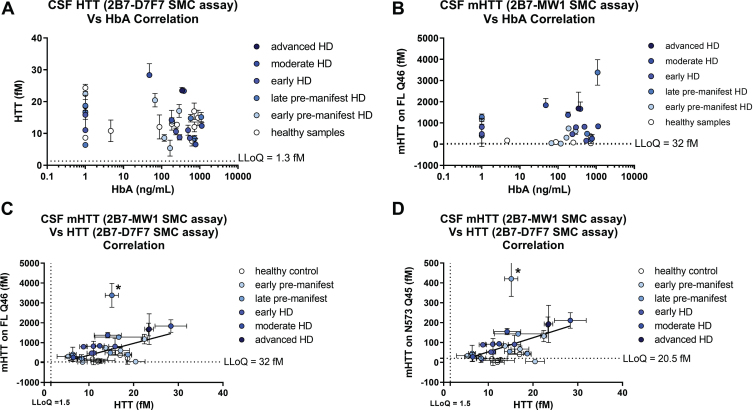
Correlations of CHDI_HTT_143 (2B7-D7F7) SMC assay HTT and CHDI_HTT_040 (2B7-MW1) SMC assay mHTT levels across HD disease stages. Due to non-Gaussian distribution of both HTT and mHTT levels as determined by the Shapiro-Wilk test, the correlation tests were carried out by a non-parametric Spearman correlation test. All samples are reported as the average and standard deviation of three technical replicates and concentrations are relative quantitative values. A) Correlation between CHDI_HTT_143 (2B7-D7F7) SMC assay HTT and HbA: *p* = 0.119, *r* = –0.26, 95% confidence interval = –0.55 to 0.08. B) Correlation between mHTT and HbA: *p* = 0.98, *r* = 0.004, 95% confidence interval = –0.37 to 0.37. C) Correlation between mHTT (as calculated against the FL Q46 HTT calibrator) and CHDI_HTT_143 (2B7-D7F7) SMC assay HTT levels in human CSF: *p* = 0.01, *r* = 0.47, 95% confidence interval = 0.12 to 0.72. Asterisks in C and D indicate a participant that was excluded from the correlation as an outlier. D) Correlation between mHTT (as calculated against the N573 Q45 HTT calibrator) and CHDI_HTT_143 (2B7-D7F7) SMC assay HTT levels in human CSF: *p* = 0.0047, *r* = 0.51, 95% confidence interval = 0.16 to 0.74. Note that when comparing the slopes of the two correlations, the y-axis of (C) and (D) are one order of magnitude different.

Next, we determined the HbA content for the 30 participant CSF samples and confirmed that the established HbA threshold of 2μg/mL is valid for the CHDI_HTT_143 (2B7-D7F7) SMC HTT assay and the CHDI_HTT_040 (2B7-MW1) SMC mHTT assay as we did not observe any correlation between HbA levels and either the CHDI_HTT_143 (2B7-D7F7) SMC assay HTT or the CHDI_HTT_040 (2B7-MW1) SMC assay mHTT levels in the tested CSF samples (Fig. 7A, B).

Finally, we set out to see if there was a correlation of mHTT levels (as calculated by either the FL Q46 HTT calibrator or the N573 Q45 HTT calibrator) and the CHDI_HTT_143 (2B7-D7F7) SMC assay HTT levels (as calculated against the FL Q46 HTT calibrator) and found that a correlation does exist (*p* = 0.01 and *p* = 0.0047, Fig. 7C and 7D, respectively). The slope of the regression line for this correlation is rather high (approximately 60 for the FL Q46 HTT and 8 for the N573 Q45 HTT calculations) due to the relative quantitative nature of mHTT. Longitudinal studies will be required to understand the extent of the contribution of mHTT in the measured CHDI_HTT_143 (2B7-D7F7) SMC assay HTT signal.

## DISCUSSION

This report describes the qualification of an ultrasensitive immunoassay employing the monoclonal antibody pair of 2B7 and D7F7 on the SMC platform for the measurement of HTT protein in a polyglutamine length-independent manner in the CSF of both HD and non-HD research participants. This assay, CHDI_HTT_143 (2B7-D7F7), is able to quantify the combined amounts of mutant (expanded) HTT and non-expanded wildtype HTT in human biosamples. We demonstrate that this assay is selective for HTT as measured by the CHDI_HTT_143 (2B7-D7F7) SMC assay, with a lower limit of quantification (LLoQ) of 1.3 fM for the full-length mHTT (1-3144) Q46 protein. Additionally, full length HTT protein variants bearing different polyglutamine lengths elicited equivalent detection signals, demonstrating the assay’s polyglutamine-length independency. The large fragment HTT (1-573) protein (N573, also designated as N548) was not detected by this assay due to the absence of the epitope recognized by the D7F7 detection antibody targeting the domain surrounding proline 1220 of HTT. One consequence is that the present assay does not account for some forms of HTT including exon1, certain proteolytic fragments, oligomers, and aggregates, hence for clarification the definition of the this HTT assay as we described it in this work is the soluble polyglutamine length-independent HTT (mHTT and non-expanded wild type HTT combined) protein detected, as confined by the capture and detection antibody epitopes being the HTT N-terminus and the HTT domain surrounding Pro1220. The CHDI_HTT_143 (2B7-D7F7) SMC assay has been shown to be specific, sensitive, and suitable for the relative-quantitation of HTT (mHTT and non-expanded wild type HTT combined) levels in human CSF samples. Additionally, this CHDI_HTT_143 (2B7-D7F7) SMC assay requires only 90μL of HD participant CSF, as opposed to the 135μL of CSF as required by the CHDI_HTT_040 (2B7-MW1) SMC mHTT assay. Higher CSF volumes tested in the SMC CHDI_HTT_143 (2B7-D7F7) SMC assay displayed an absence of dilution linearity, therefore should not be used.

The CHDI_HTT_143 (2B7-D7F7) assay is compliant with FDA and EMA acceptance criteria for ligand binding assays, but as expected for ultra-sensitive assays it does display some degree of variability, thus we highly recommended that samples are analyzed in technical triplicates. Additionally, in the absence of a test/re-test assessment due to the limited availability of human HD CSF, the best results for this type of analysis are obtained by testing all samples for one participant in the same assay plate and run, provided that the biosample is stable for the interval being tested. We did not conduct stability assessment of frozen CSF in this particular qualification work, again due to the limited availability of HD CSF. The analyte stability in CSF for the CHDI_HTT_143 (2B7-D7F7) SMC assay should be investigated in a future assay validation work.

The goal of this work was to qualify a polyglutamine length-independent (mHTT and non-expanded wild type HTT combined detection) HTT SMC assay (here the CHDI_HTT_143 (2B7-D7F7) SMC assay) that could be used together with the established CHDI_HTT_040 (2B7-MW1) mHTT SMC assay to better understand, and perhaps mitigate, the known relative-quantitative issues of the mHTT assay in regards to the sensitivity to the polyglutamine-expansion length and the form or length of the HTT protein present in the biosample. Furthermore, the CHDI_HTT_143 (2B7-D7F7) SMC assay should facilitate the interpretation of the results of allele-selective mHTT-lowering interventions. And finally, although the present work focuses on CSF as a brain surrogate tissue, we anticipate that the CHDI_HTT_143 (2B7-D7F7) SMC assay may be very useful as a pharmacodynamic readout for peripheral HTT modulation detection in the clinic, including in Phase I trials in healthy volunteers.

The current analysis of CHDI_HTT_143 (2B7-D7F7) SMC assay HTT levels in CSF samples from control and HD participants revealed no correlation with disease stage, which is in contrast to the previously reported increase for mHTT with disease stage [5]. This observation may require a larger study with additional advanced HD research participants to ensure that we did not underestimate the CHDI_HTT_143 (2B7-D7F7) SMC assay HTT levels at advanced HD stages due to the limited number of such samples available for the present study. In addition, in a combined data analysis, we found that CHDI_HTT_143 (2B7-D7F7) SMC assay HTT levels in control participant CSF samples to be marginally lower than CHDI_HTT_143 (2B7-D7F7) SMC assay HTT levels in HD participant CSF samples. However, when we compared CHDI_HTT_040 (2B7-MW1) SMC assay mHTT and CHDI_HTT_143 (2B7-D7F7) SMC HTT levels in the CSF from the same HD research participants we found that they correlate well. In the present dataset, we observed what we have previously called the “paradox of mHTT levels [6, 9]”, in that mHTT concentrations are observed to be higher than that of the calculated polyglutamine length-independent (mHTT and non-expanded wild type HTT combined) HTT concentrations. This may be due to the fact that the mHTT assay is sensitive to the polyglutamine expansion length as well as the form or size of the HTT protein present in the biosample with respect to the recombinant mHTT protein used as the assay calibrator. This results in a calculated mHTT concentration that cannot be referred to as absolute, and therefore cannot be compared among research participants.

Nevertheless, even despite the small sample size of the research participants, which may limit the interpretation of these results, the preliminary observation that there is a correlation between CHDI_HTT_040 (2B7-MW1) SMC assay mHTT and CHDI_HTT_143 (2B7-D7F7) SMC assay HTT levels in HD research participant CSF samples but at the same time not in the CHDI_HTT_143 (2B7-D7F7) SMC assay HTT levels across disease stages which warrants further investigation into the biology of HTT proteoforms in CSF. We postulate that this observation could also be influenced by the specific epitopes of the antibodies being employed. Specifically, in the CHDI_HTT_143 (2B7-D7F7) SMC HTT assay, the detection antibody D7F7 binds farther towards the C-terminus of HTT at proline 1220 and is thus likely to quantify only the full-length and larger soluble fragmented HTT proteins in a polyglutamine length-independent manner, and will not quantify N-terminal fragments shorter than proline 1220. Therefore, it should be noted that this CHDI_HTT_143 (2B7-D7F7) SMCT assay will not detect the reported exon 1 splice variant [24] if present in the biosample.

In conclusion, we demonstrate that we can detect and quantitate HTT in a polyglutamine length-independent manner (mHTT and non-expanded wild type HTT combined detection) in human control and HD CSF samples and demonstrate that this ultrasensitive assay can track the dynamics of HTT levels potentially contributing to the outcome of clinical interventions that aim to lower HTT. Future clinical and preclinical studies will benefit from utilization of this CHDI_HTT_143 (2B7-D7F7) SMC HTT assay in combination with other HTT assays.
